# High jugular bulb in patients with non‐thrombotic internal jugular venous and transverse sinus stenosis: Clues to pathogenesis

**DOI:** 10.1111/cns.14424

**Published:** 2023-08-29

**Authors:** Zhongao Wang, Chaitu Dandu, Yibing Guo, Meini Gao, Zixiang Wang, Duo Lan, Liqun Pan, Da Zhou, Yuchuan Ding, Xunming Ji, Ran Meng

**Affiliations:** ^1^ Department of Neurology, National Center for Neurological Disorders, Xuanwu Hospital Capital Medical University Beijing China; ^2^ Advanced Center of Stroke Beijing Institute for Brain Disorders Beijing China; ^3^ Department of Neurosurgery Wayne State University School of Medicine Detroit Michigan USA; ^4^ Department of General Practice Shuangfengsi Central Health Center Chengde Hebei China; ^5^ Department of Neurosurgery, Xuanwu Hospital Capital Medical University Beijing China

**Keywords:** hemodynamics, high jugular bulb, internal jugular venous stenosis, pathogenesis, transverse sinus stenosis

## Abstract

**Aims:**

Conventional theories for jugular bulb (JB) formation are insufficient to explain the high proportion of high JB in adult patients. We aimed to study features of high JB in patients with non‐thrombotic internal jugular venous stenosis (IJVS) and/or transverse sinus stenosis (TSS) to explore the pathogenesis of high JB formation.

**Methods:**

We retrospectively enrolled consecutive patients with the diagnosis of non‐thrombotic IJVS and/or TSS. The relationship between IJVS and/or TSS and high JB was explored. Logistic regression analysis was performed to identify potential independent risk factors for high JB.

**Results:**

A total of 228 patients were included in the final analyses. The proportions of IJVS, dominant‐side IJVS, and non‐TSS in dominant‐side high JB subgroup were higher than those in nondominant‐side high JB subgroup (83.3% vs. 62.5%, *p* < 0.001; 72.2% vs. 18.3%, *p* < 0.001; 43.5% vs. 29.2%, *p* = 0.02). Heights of JBs on dominant sides in IJVS subgroup and non‐TSS subgroup were higher than those in non‐IJVS subgroup and TSS subgroup (12.93 ± 2.57 mm vs. 11.21 ± 2.76 mm, *p* < 0.001; 12.66 ± 2.71 mm vs. 11.34 ± 2.73 mm, *p* = 0.003). Multivariate logistic regression indicated an independent association between dominant‐side IJVS and dominant‐side high JB (odds ratio, 29.40; 95% confidence interval, 11.04–78.30; *p* < 0.001).

**Conclusion:**

IJVS and asymmetric transverse sinus were independently and positively associated with high JB, especially dominant‐side IJVS with dominant‐side high JB, indicating a potential hemodynamic relationship between IJVS and high JB formation. Conversely, TTS might impede high JB formation.

## Graphical abstract

IJVS is independently and positively associated with high JB formation, especially dominant‐side IJVS with dominant‐side high JB, indicating that IJVS downstream might promote the elevation of JB by generating an ascending venous hydraulic force ipsilaterally. Conversely, TSS upstream of JB might impede high JB formation.

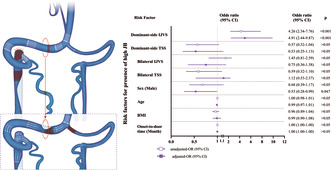

## 1 INTRODUCTION

The jugular bulb (JB) is the conjunction of intra‐ and extracranial venous system and plays an important role in cerebral venous outflow. It receives the venous blood from sigmoid sinus directly at a nearly right‐angled turn and bears the brunt of blood flow.^1^ The high JB and its related variants might be associated with a series of diseases, but its etiology is still not fully understood by the lack of sufficient clinical evidence.^1^, ^2^, ^3^ One postulated mechanism for the development of JB was the switch to an erect posture around two years of age, and ascending pulse waves might be formed to enlarge JBs after the posture change. Variations during this process might cause asymptomatic high JBs in the normal population.^4^, ^5^

However, the reported incidence of high JB in patients with symptomatic ear diseases was significantly higher than that in the normal population.^3^, ^6^, ^7^, ^8^ Conventional theories appeared insufficient in such a high proportion of adult patients, which posited that JBs remained relatively stable in adulthood.^4^ Meanwhile, the dominant drainage of cerebral venous outflow is paraspinal collateral veins in the erect position as IJVs collapse.^9^, ^10^ And venous valves in IJVs can prevent the venous reflex by central venous pressure, indicating an attenuated turbulent flow at JB. Consequently, other mechanisms might exist to drive the elevation of JB, as it is surrounded by firm osseous structures. Previous studies revealed that patients with IJVS and/or TSS could have comorbid high JB with symptoms such as tinnitus, head noise, and hearing impairment, which could partly be attributed to the high JB and its encroachment of the cochlea.^2^, ^11^, ^12^, ^13^, ^14^ These clues suggested a possible relationship between IJVS and/or TSS and high JB formation, which was not fully appreciated previously.

Theoretically, IJVS could obstruct cerebral venous outflow downstream of JB, which might increase the venous pressure at JB and create an ascending force to the dome of ipsilateral JB directly, being independent of body postures. The bones surrounding JBs might be thinned by the ascending force over the long term, resulting in high JB formation.^15^, ^16^ The computational fluid dynamics study indeed proved the pronounced vortex in the elevated JB but failed to consider the coexisting IJVS downstream of JB.^17^ On the other hand, for patients with TSS, the trans‐stenosis pressure gradient could decrease the venous pressure and alleviate the turbulent flow at JB, which might function as an inhibitory factor for high JB formation.^18^, ^19^, ^20^ Accordingly, we assumed that IJVS could promote high JB formation by generating an ascending force to the JB, and TSS might impede high JB formation by alleviating the venous pressure at JB. Therefore, this study aimed to investigate correlations between non‐thrombotic IJVS and/or TSS and high JB with head‐and‐neck computed tomography venography (CTV) examinations considering the dominant cerebral venous drainage. We would also analyze the relationship by adjusting for confounders, which was not commonly performed in previous studies but was important in cerebral venous diseases.

## 2 METHODS

This study retrospectively analyzed consecutive patients with a first‐time diagnosis of non‐thrombotic IJVS and/or TSS from the Neurology department in Xuanwu Hospital, Capital Medical University between January 2019 and December 2021. The study design was approved by the Institutional Ethic Committee of Xuanwu Hospital, Capital Medical University following the Declaration of Helsinki. We have obtained written informed consent from the patients who took part in our regular follow‐up in the outpatient clinic or via mailing after telephone contact. The need for informed consent was waived by the Institutional Ethic Committee for cases who were unable to be contacted.

The inclusion criteria were as follows: (a) a first‐time diagnosis of non‐thrombotic IJVS and/or TSS by head‐and‐neck magnetic resonance venography (MRV) or CTV and brain MRI in our hospital; (b) abnormally dilated vertebral veins in patients with IJVS; (c) images of CTV for imaging assessment; (d) no restriction about age or gender. The exclusion criteria included (a) venous valve insufficiency or venous reflux of IJV; (b) other cerebral vascular disorders, such as definitive intracranial or extracranial arterial stenosis, cerebral venous thrombosis, and arteriovenous fistula; (c) superior vena cava syndrome or cardiac dysfunction, which might interfere with cerebral venous drainage; (d) histories of cerebral infarction or intracranial hemorrhage; (e) abnormal liver or renal function, or neoplastic disease; (f) incomplete clinical or radiographic data.

### 2.1 Assessment

As previously defined, a high JB was diagnosed if it extended above the basal turn of the cochlea in CTV imaging.^2^, ^21^, ^22^, ^23^ The height of JB was measured in the sagittal view of CTV and was defined as the vertical distance from the inferior margin of jugular foramen to the highest point of JB dome.^24^ The IJVS was defined if the minimum cross‐sectional area at the narrow segment of IJV was <50% of the normal cross‐sectional area at the proximal adjacent segment of IJV.^25^ The abnormally dilated vertebral vein was diagnosed if the maximal cross‐sectional area of the vertebral vein was ≥25% of the cross‐sectional area at the same segment of the adjacent IJV.^25^ The TSS was defined if the minimum cross‐sectional area at the narrow segment of transverse sinus was <50% of the normal cross‐sectional area at the proximal adjacent segment of transverse sinus.^26^ The asymmetric transverse sinus (unilateral dominant cerebral venous drainage) was diagnosed if the difference between the maximal cross‐sectional areas of bilateral transverse sinuses was ≥50% of the maximal cross‐sectional area at the smaller transverse sinus.^4^, ^27^

The assessment of CTV images was conducted by two independent investigators retrospectively, who were blinded to general information and clinical data of patients, with eight years (D.Z.) and 11 years (R.M.) of experience in diagnosing cerebral venous diseases. The axial source images and multiplanar reformatted images in the sagittal and coronal views were available for precise evaluation. Any disagreements were finally resolved by a third investigator with 12 years of experience in diagnosis (XM.J.).

### 2.2 Statistical analysis

Normally distributed continuous variables were presented as mean ± standard deviation (SD) and non‐normally distributed continuous variables as median (IQR). Categorical variables were described as number (percentage). The normality of distribution was visually tested with histograms and normal probability plots (Q–Q plots). The intraclass correlation coefficients (ICCs) were computed to assess the interrater reliability in measuring heights of JBs. Reliability was classified as excellent (>0.90), good (0.75–0.90), moderate (0.50–0.75), and poor (<0.50). Once the ICCs were deemed excellent, the two readers' results were averaged to get the final heights of JBs. The Pearson chi‐square or Fisher's exact test was used to compare categorical data. Intergroup differences in continuous variables were tested via independent sample *t*‐test or Mann–Whitney *U* test. Multivariable binary logistic regression models were constructed to identify potential independent risk factors for features of high JB. Three features of high JB were considered as dependent variables, including the presence or absence of high JB, with or without dominant‐side high JB, and unilateral or bilateral high JB. Covariates were selected based on a priori hypothesis and/or *p*‐value < 0.2 in the univariate analysis. The covariates entered into final models included IJVS, TSS, asymmetric transverse sinus, sides of stenosis, sex, age, body mass index (BMI), and onset‐to‐door time, as appropriate. Statistical significance was determined when *p*‐value < 0.05 (two‐sided). The statistical analyses were carried out using SPSS software for Windows, Version 21.0 (IBM Corp., Armonk, NY, USA). The statistical power analysis was conducted with the PASS software, Version 11.0 (NCSS, LLC, Kaysville, Utah, USA).

## 3 RESULTS

### 3.1 Clinical presentations

**FIGURE 1:**
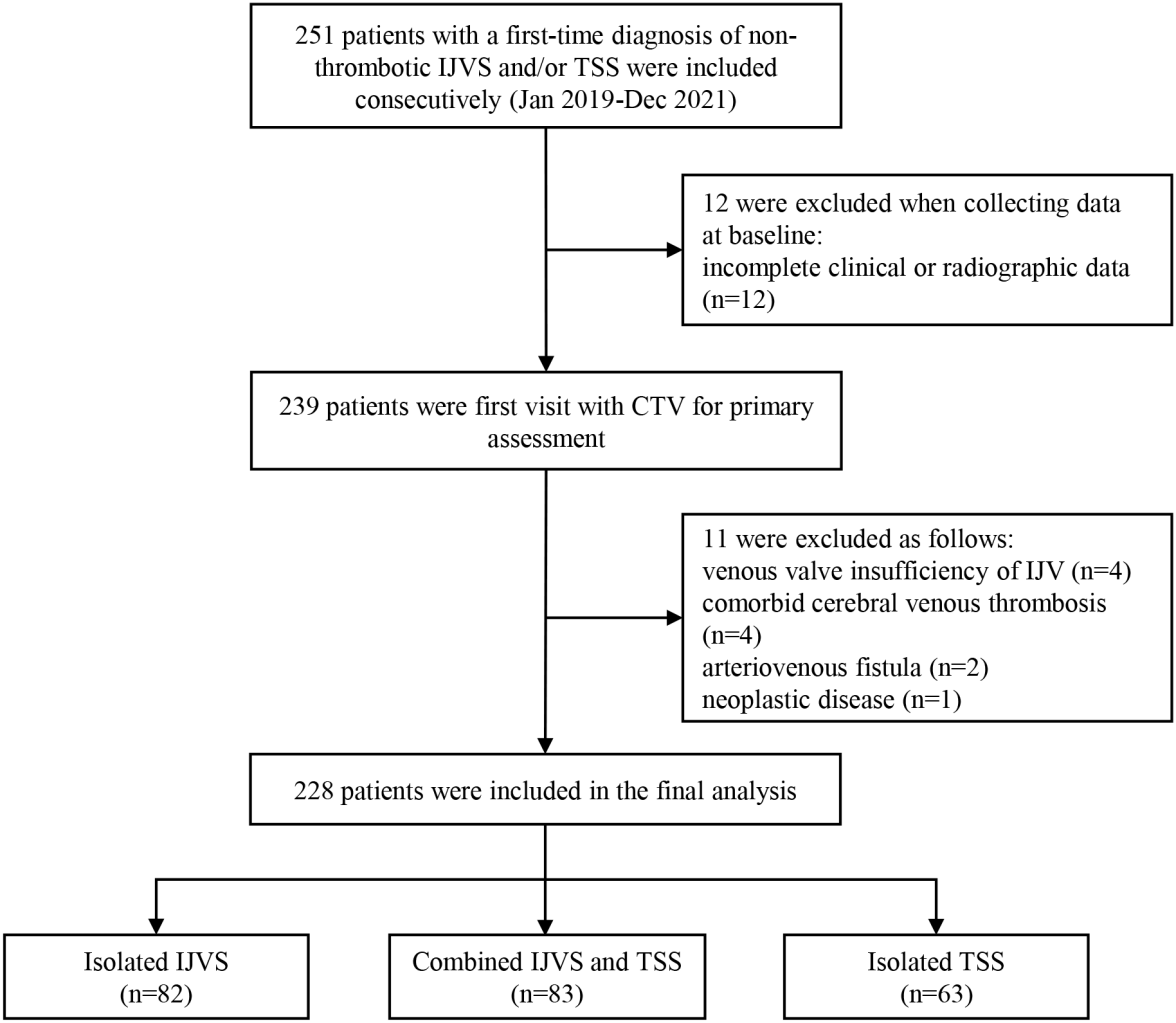
Patient flow chart.

In total, 251 patients with a first‐time diagnosis of non‐thrombotic IJVS and/or TSS were consecutively included. After initial assessments, 23 patients were excluded. Among them, 12 patients had incomplete clinical or radiographic data; four patients had venous valve insufficiency of IJV; four patients had comorbid cerebral venous thrombosis; two patients had arteriovenous fistula; and one patient had neoplastic disease. The enrollment process is illustrated in Figure 1. Accordingly, 228 patients were included in the final analyses with 85 males and 143 females. The average age at diagnosis was 50.22 ± 14.53 years. The median onset‐to‐door time was 18.00 (IQR, 6–60) months. The average BMI was 24.71 ± 3.40 kg/m^2^. The main symptoms included headache (45.6%), tinnitus (43.0%), head noise (43.0%), sleep disturbance (41.2%), dizziness (37.3%), visual impairment (35.1%), and hearing impairment (16.2%). Details are available in Table 1. No significant correlation was found between clinical features of these patients and the presence of high JB, which is presented in Table S1.

**TABLE 1:** Clinical features of patients with non‐thrombotic IJVS and/or TSS.

Clinical features	
Demographics	
No. of patients	228
Gender (male/female)	85/143
Age (years) (Mean ± SD)	50.22 ± 14.53
Onset‐to‐door time (months) (Median, IQR)	18.00 (IQR, 6–60)
BMI (kg/m^2^)	24.71 ± 3.40
Clinical symptoms (No., %) *n* = 228	
Headache	104 (45.6)
Tinnitus	98 (43.0)
Head noise	98 (43.0)
Sleep disturbance	94 (41.2)
Dizziness	85 (37.3)
Visual impairment	80 (35.1)
Hearing impairment	37 (16.2)
Dry or puffy eyes	28 (12.3)
Vertigo	18 (7.9)
Double vision	12 (5.3)

Abbreviations: BMI, body mass index; IJVS, internal jugular venous stenosis; TSS, transverse sinus stenosis; SD, standard deviation; IQR, interquartile range.

### 3.2 Imaging presentations

Among the 228 patients, 83 (36.4%) had combined IJVS and TSS, 82 (36.0%) had isolated IJVS, and 63 (27.6%) had isolated TSS. The asymmetric transverse sinus was present in 174 (76.3%) patients. IJVS was on the dominant side in 100 (43.9%) patients, and TSS was on the dominant side in 61 (26.8%) patients. Of all patients, 142 (62.3%) had concomitant high JB with 32 (14.0%) being bilateral and 110 (48.2%) being unilateral. The high JB was on the dominant side in 108 (47.4%) patients, on the right side in 112 (49.1%) patients, and on the left side in 62 (27.2%) patients. To assure the rationale for this sample size, we used two‐tailed tests at a significance level of 0.05. In summary, the estimated statistical power reached more than 0.90 when considering the proportions of high JB and dominant‐side high JB in each group as the effect sizes. So, the sample size is sufficient to power the significant differences (at least 0.80) in this study statistically.

### 3.3 Correlation between non‐thrombotic IJVS and/or TSS and high JB

**FIGURE 2:**
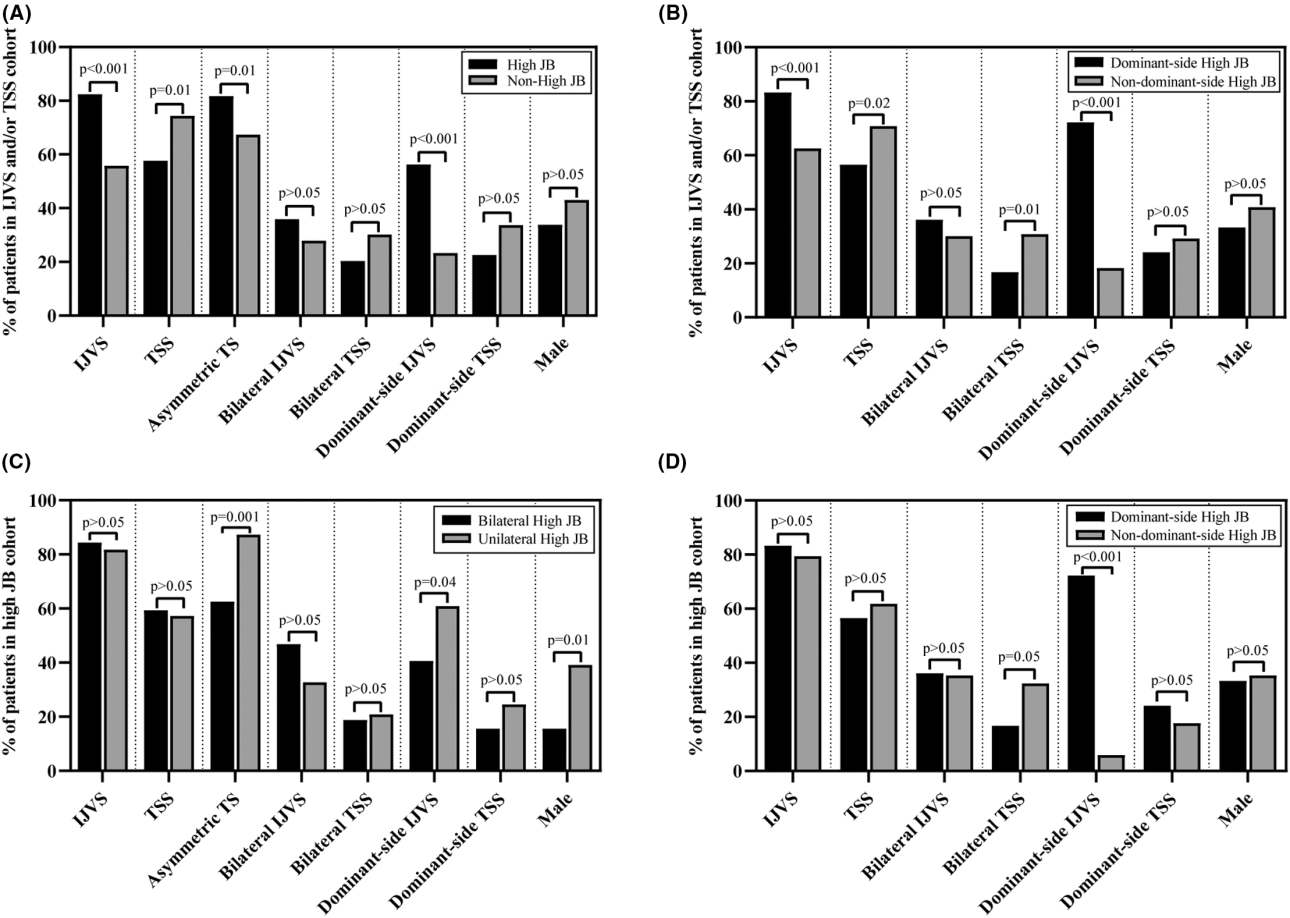
Correlations between the features of high JB and the features of IJVS or TSS. Panels A, B: the proportion of patients with different features of IJVS or TSS in the non‐thrombotic IJVS and/or TSS cohort. (A) Comparison between high JB subgroup and non‐high JB subgroup. (B) Comparison between dominant‐side high JB subgroup and nondominant‐side high JB subgroup. Panels C, D: the proportion of patients with different features of IJVS or TSS in the subset of patients with high JB exclusively. (C) Comparison between bilateral high JB subgroup and unilateral high JB subgroup. (D) Comparison between dominant‐side high JB subgroup and nondominant‐side high JB subgroup. IJVS, internal jugular venous stenosis; JB, jugular bulb; TS, transverse sinus; TSS, transverse sinus stenosis.

As shown in Figure 2 (panel A), in the IJVS and/or TSS cohort, patients were divided into two subgroups according to the presence of high JB. Among them, the proportions of IJVS, asymmetric transverse sinus, and dominant‐side IJVS in high JB subgroup were higher than those in non‐high JB subgroup (82.4% vs. 55.8%, *p* < 0.001; 81.7% vs. 67.4%, *p* = 0.01; 56.3% vs. 23.3%, *p* < 0.001, respectively). The proportion of TSS in high JB subgroup was lower than that in non‐high JB subgroup (57.7% vs. 74.4%, *p* = 0.01). Additionally, the patients were divided into two subgroups according to the presence of dominant‐side high JB (Figure 2, panel B). Interestingly, the proportions of IJVS and dominant‐side IJVS in dominant‐side high JB subgroup were still higher than those in nondominant‐side high JB subgroup (83.3% vs. 62.5%, *p* < 0.001; 72.2% vs. 18.3%, *p* < 0.001, respectively). And the proportions of TSS and bilateral TSS in dominant‐side high JB subgroup were lower than those in nondominant‐side high JB subgroup (56.5% vs. 70.8%, *p* = 0.02; 16.7% vs. 30.8%, *p* = 0.01, respectively).

To further verify the results, we retested the relationships in the subset of patients with high JB exclusively. As shown in Figure 2 (panel C), the patients with high JB were divided into two subgroups according to the presence of bilateral high JB. The proportions of asymmetric transverse sinus and dominant‐side IJVS in bilateral high JB subgroup were lower than those in unilateral high JB subgroup (62.5% vs. 87.3%, *p* = 0.001; 40.6% vs. 60.9%, *p* = 0.04, respectively). The proportion of males in bilateral high JB subgroup was lower than that in unilateral high JB subgroup (15.6% vs. 39.1%, *p* = 0.01). In addition, as illustrated in Figure 2 (panel D), the patients with high JB were divided into two subgroups according to the presence of dominant‐side high JB. The proportion of dominant‐side IJVS in dominant‐side high JB subgroup was higher than that in nondominant‐side high JB subgroup (72.2% vs. 5.9%, *p* < 0.001). And the proportion of bilateral TSS in dominant‐side high JB subgroup was lower than that in nondominant‐side high JB subgroup (16.7% vs. 32.4%, *p* = 0.05).

**FIGURE 3:**
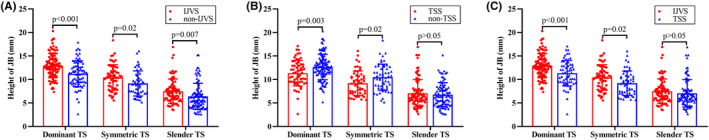
The heights of JBs on the side of dominant, symmetric and slender transverse sinuses. (A) The heights of JBs in IJVS subgroup vs. no‐IJVS subgroup. (B) The heights of JBs in TSS subgroup vs. no‐TSS subgroup. (C) The heights of JBs in IJVS subgroup vs. TSS subgroup. IJVS, internal jugular venous stenosis; JB, jugular bulb; TS, transverse sinus; TSS, transverse sinus stenosis.

The transverse sinuses were divided into three subtypes according to their dominance, including dominant, symmetric, and slender transverse sinuses. The ICC for measuring heights of JBs was 0.99 (95% CI 0.99 to 0.99). The heights of JBs on the side of dominant transverse sinus and symmetric transverse sinus in IJVS subgroup were higher than those in non‐IJVS subgroup (12.93 ± 2.57 mm vs. 11.21 ± 2.76 mm, *p* < 0.001; 10.43 ± 2.71 mm vs. 9.16 ± 2.67 mm, *p* = 0.02, respectively). Details are revealed in Figure 3 (panel A). The heights of JBs on the side of dominant transverse sinus and symmetric transverse sinus in TSS subgroup were lower than those in non‐TSS subgroup (11.34 ± 2.73 mm vs. 12.66 ± 2.71 mm, *p* = 0.003; 9.20 ± 2.59 mm vs. 10.48 ± 2.78 mm, *p* = 0.02, respectively). Details are given in Figure 3 (panel B).

**FIGURE 4:**
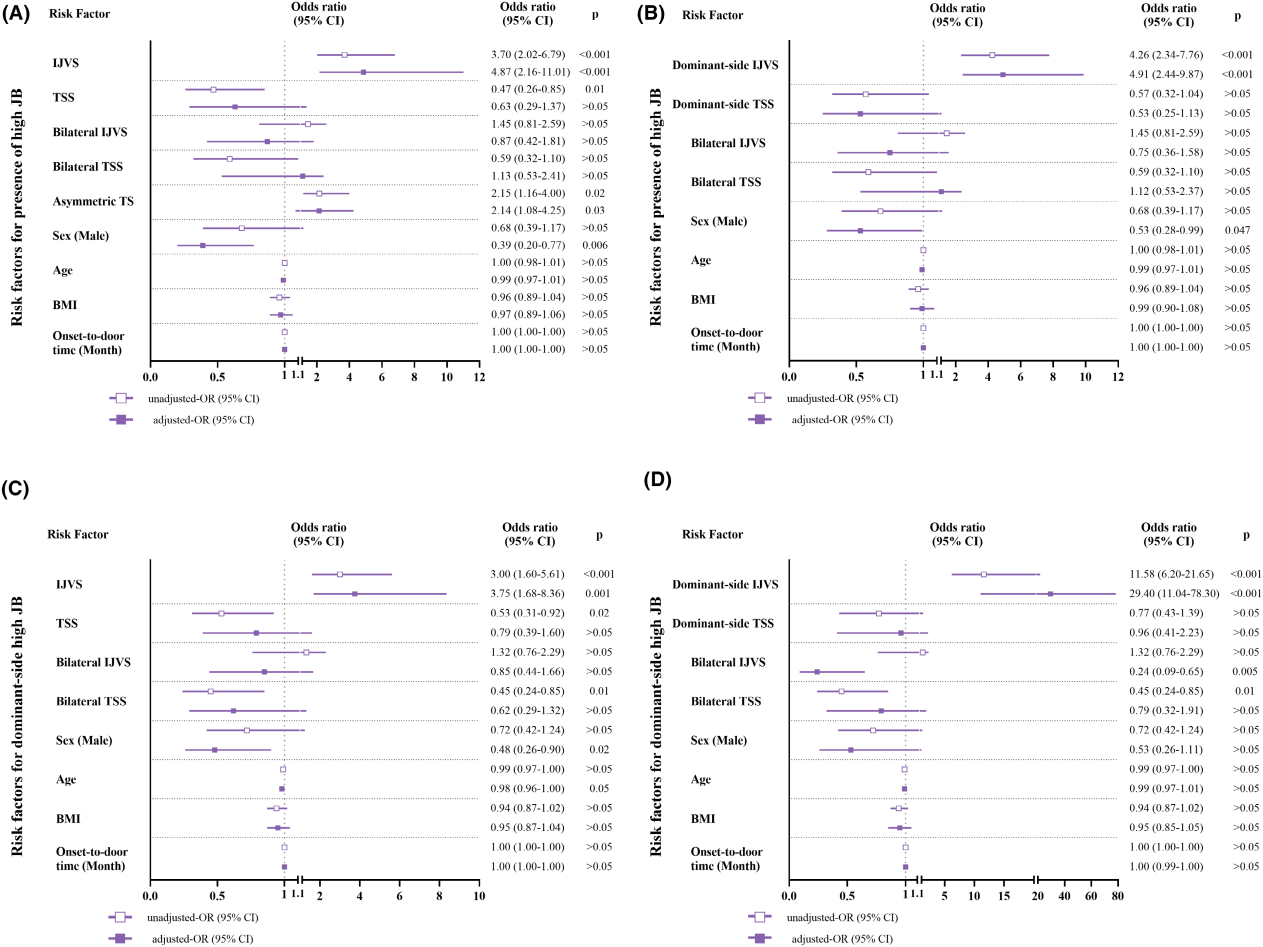
Forest plot of the potential risk factors for the features of high JB in patients with non‐thrombotic IJVS and/or TSS. Panels (A, B): potential risk factors for the presence of high JB. Panels (C, D): potential risk factors for the presence of dominant‐side high JB. BMI, body mass index; CI, confidence interval; IJVS, internal jugular venous stenosis; JB, jugular bulb; OR, odds ratio; TS, transverse sinus; TSS, transverse sinus stenosis.

As illustrated in Figure 4, we constructed multivariate logistic regression models in patients with non‐thrombotic IJVS and/or TSS. Firstly, we analyzed the factors associated with the presence of high JB (Figure 4, panels A, B). After adjusting for confounders, both IJVS (*p* < 0.001) and asymmetric transverse sinus (*p* = 0.03) were associated with the presence of high JB, and males were less likely to have high JB than females (*p* = 0.006). Details are given in Figure 4 (panel A). Interestingly, when we combined IJVS and asymmetric transverse sinus into the dominant‐side IJVS, the odds of high JB increased (Figure 4, panel B). Furthermore, as shown in Figure 4 (panel D), we tested whether the side of IJVS was independently correlated with the side of high JB. After adjusting for confounders, dominant‐side IJVS was independently correlated with dominant‐side high JB (*p* < 0.001), showing increased odds compared to IJVS alone (Figure 4, panels C, D). TSS and bilateral TSS seemed to reduce the odds of dominant‐side high JB, which were insignificant after adjustment. In contrast, male (*p* = 0.02) and bilateral IJVS (*p* = 0.005) reduced the odds of dominant‐side high JB significantly after adjustment (Figure 4, panels C, D).

As shown in Figure S1, we focused on the subset of patients with high JB exclusively and analyzed factors associated with the features of high JB. As displayed in Figure S1 (panel A), dominant‐side IJVS (*p* < 0.001) showed significantly higher odds of dominant‐side high JB compared to nondominant‐side IJVS after adjustment, and bilateral IJVS (*p* = 0.01) showed lower odds. Additionally, as for the bilateral or unilateral high JB, bilateral IJVS (*p* = 0.02) increased the odds of bilateral high JB after adjustment while dominant‐side IJVS (*p* = 0.008) and male (*p* = 0.006) were independently associated with reduced odds of bilateral high JB (Figure S1, panel B).

## 4 DISCUSSION

In this study, we found that IJVS was independently and positively associated with high JB, especially dominant‐side IJVS with dominant‐side high JB. In contrast, TSS might inhibit high JB formation. In univariate analysis, the proportions of IJVS, asymmetric transverse sinus, and dominant‐side IJVS in high JB subgroup were higher than those in non‐high JB subgroup. Similarly, the proportions of IJVS and dominant‐side IJVS in dominant‐side high JB subgroup were higher than those in nondominant‐side high JB subgroup. The correlation could also be proved in patients with high JB exclusively. The results implied that IJVS and asymmetric transverse sinus might promote high JB formation. Their combined effect, the dominant‐side IJVS downstream of JB, might primarily promote high JB formation ipsilaterally, revealing a direct blood flow impingement in the pathogenesis of high JB. Additionally, the proportions of TSS in high JB subgroup and dominant‐side high JB subgroup were lower than those in non‐high JB subgroup and nondominant‐side high JB subgroup, showing that TSS might impede high JB formation. Moreover, when measuring heights of JBs directly on sides of dominant and symmetric transverse sinuses, the heights of JBs with IJVS and with non‐TSS were higher than those with non‐IJVS and with TSS, respectively. The results further proved that IJVS might promote and TSS might inhibit high JB formation.

We tested the aforementioned results by adjusting for confounders. In multivariate analysis, IJVS and asymmetric transverse sinus were independently associated with the presence of high JB. And their combined effect, the dominant‐side IJVS, could increase the odds of high JB further, indicating a mutual reinforcement in hemodynamic changes (Figure 4, panels A, B). When considering the sides of IJVS and high JB simultaneously, dominant‐side IJVS could even increase the odds of dominant‐side high JB to 29.40 (Figure 4, panel D), revealing that high JB formation was most likely the result of IJVS downstream of the dominant side, and the subsequent ascending force to the dome of ipsilateral JB directly. This effect was strengthened when being analyzed in patients with high JB exclusively (Figure S1, panel A). Although TSS was not as stable as IJVS to be independently associated with high JB in logistic regression, it displayed a trend to reduce the odds of high JB and dominant‐side high JB after adjustment, implying the tendency of TSS to resist high JB formation. Meanwhile, the influence of IJVS and TSS on high JB could be intricate by a group of confounders such as stenosis degree, distance to JB, and temporal orders of stenosis formation, which required a long‐term follow‐up to monitor the changes dynamically.

From an embryonic and developmental standpoint, the JB originated from a narrowed connection between sigmoid sinus and IJV, named jugular sinus. It was not enlarged until two years of age or older when a bulb‐like structure could be discovered in postmortem and radiologic examinations. And its size remained relatively stable in adulthood.^4^, ^5^, ^6^ The reason for its development postnatally was linked to the switch to erect posture around the age, and an ascending negative pulse wave, from the right atrium, towards the domes of jugular sinuses might be formed to enlarge JBs after the posture change. And variations during this process could cause the asymptomatic high JB or its related ear diseases.^21^ The reported incidence of high JB was about 6%–20% in patients for various reasons or postmortem^7^, ^21^, ^23^ and 15%–47% in patients with ear symptoms.^3^, ^8^, ^28^ In clinical practice, high JB and its relevant diverticulum or dehiscence were associated with a series of ear diseases such as pulsatile tinnitus and Meniere's disease.^1^, ^2^, ^8^ And the symptoms could be relieved after the surgical intervention.^29^, ^30^ However, some defects in the existing theories were discovered as well. Conventional theories could explain the small proportion of asymptomatic variants in the normal population, but patients with severe ear diseases had a higher incidence of high JB. And their onset age and peak prevalence age was older when the JB remained stable in size.^4^, ^6^, ^31^, ^32^ Moreover, the dominant drainage of cerebral venous outflow is paraspinal collateral veins in the erect position as IJVs collapse.^9^, ^10^ And venous valves in IJVs can prevent the venous reflex by central venous pressure. Consequently, the venous hemodynamic disturbance at JB would be markedly attenuated in the erect position by the decreased blood flow through IJVs.

**FIGURE 5:**
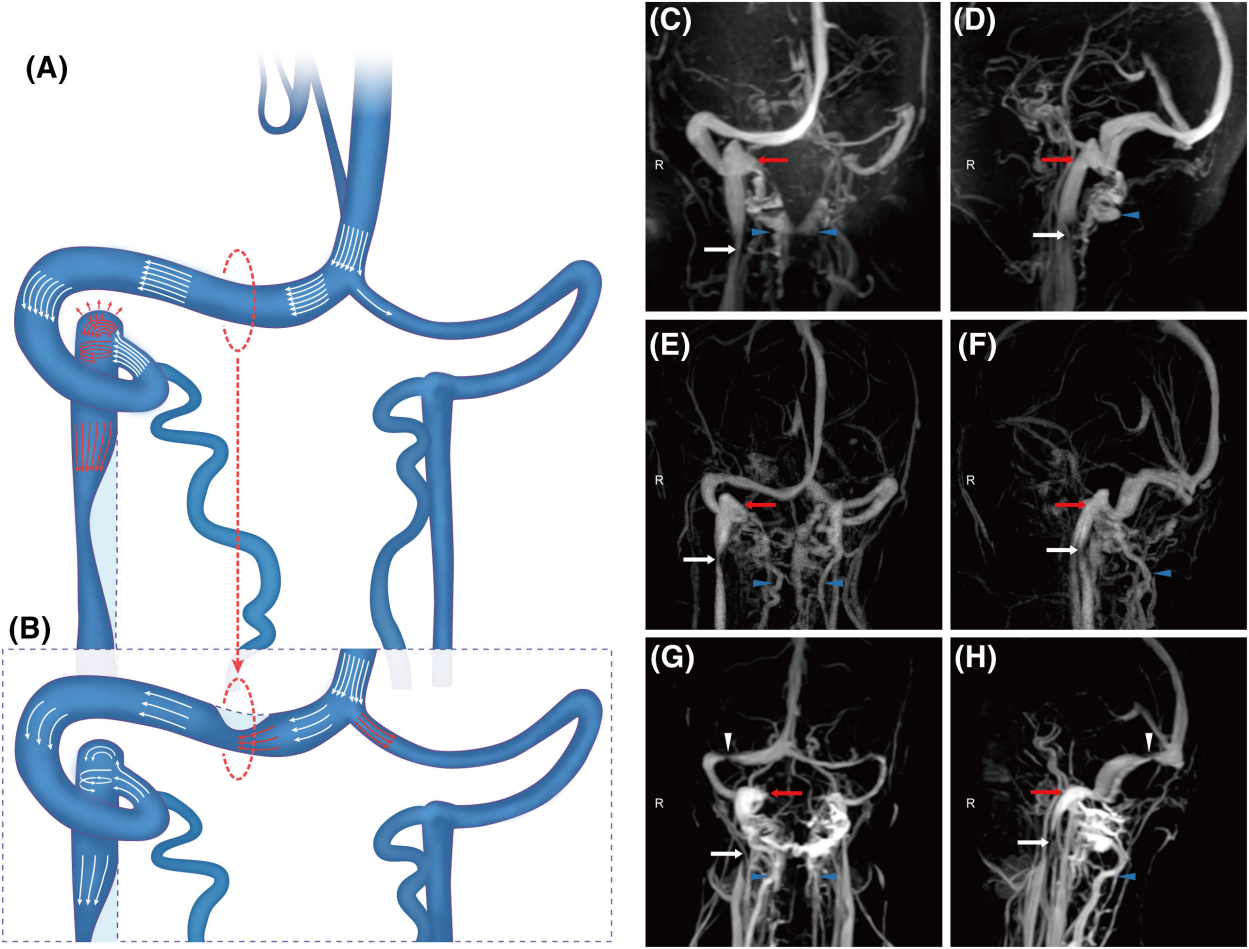
A proposed mechanism for the influence of IJVS and TSS on high JB formation. Panel (A): For patients with IJVS on the dominant side, a trans‐stenosis pressure gradient could increase the pressure downstream of JB (red arrows). The increased pressure could act as an external force to drive the elevation of JB. Panel (B): The TSS (red dotted circle) on the dominant side could decrease the blood flow and pressure at the ipsilateral JB (the sparse white arrows) by increasing the pressure upstream of TSS and shunting the cerebral venous outflow to the contralateral transverse sinus (red arrows). The decreased pressure by TSS might impede high JB formation. Panels (C–H): the images of patients with IJVS (white arrow) and/or TSS (white triangle). The abnormally dilated vertebral veins (blue arrow) indicated the insufficient cerebral venous outflow through the internal jugular vein by IJVS. A patient with IJVS and high JB (red arrow) on the dominant side (panels C, D). A patient had IJVS and high JB (red arrow) on the right side even with symmetric transverse sinus (panels E, F). A patient had IJVS and TSS on the dominant side without an elevated JB ipsilaterally (panels G, H). IJVS, internal jugular venous stenosis; JB, jugular bulb; TSS, transverse sinus stenosis.

The theory of ascending pulse waves by posture changes seems to be insufficient for high JB formation in such a high proportion of adult patients. A persistent external force is reasonable to drive the elevation of JB, as it is surrounded by firm osseous structures. As demonstrated in Figure 5, a possible explanation for the hemodynamic cause of high JB might be the IJVS, which is located upstream of venous valves. The IJVS could increase the venous pressure at JB and generate an ascending venous hydraulic force to JB directly.^16^ The bones surrounding JBs might be thinned by the ascending force over the long term, resulting in high JB formation.^15^, ^16^ And the IJVS was commonly the result of osseous compression, which could also progress gradually synchronizing with osseous changes surrounding the elevated JB.^12^, ^14^, ^25^ In this study, dominant‐side IJVS was independently and positively associated with high JB and especially the dominant‐side high JB. The dominant‐side IJV undertook the major cerebral venous drainage, whose stenosis could cause severe venous turbulence at the JB ipsilaterally.^12^, ^16^ Additionally, the incidence of asymmetric transverse sinus in this study was similar to that in the normal population, indicating that, apart from hyperdynamic venous outflow, the co‐occurrence of IJVS might be an important promotor for high JB formation.^4^, ^21^ What is more, previous studies found that ligating unilateral IJV could decrease the size of ipsilateral JB by transferring venous outflow to collateral circulation completely, and a pronounced vortex in the elevated JB was proved by the computational fluid dynamics study.^17^, ^33^, ^34^ These studies further proved that the hemodynamic changes at IJV and JB might be a major contributor to the elevation of JB.^17^, ^33^, ^34^ On the other hand, for patients with TSS, a pressure gradient was formed at the stenosis segment. At downstream of TSS, the venous pressure of ipsilateral JB was decreased to alleviate the expansion force to surrounding osseous structures.^18^, ^19^, ^20^ And the increased venous pressure upstream of TSS could shunt the venous flow to the contralateral transverse sinus, which would further decrease the venous pressure and turbulent flow at the ipsilateral JB.^35^ These combined effects of TSS might function as an inhibitory factor for high JB formation. A proposed mechanism for the influence of IJVS and TSS on high JB formation is depicted in Figure 5.

Our study had several advantages. First, we, for the first time, included IJVS as a possible impact of high JB and evaluated the lesions up‐ and downstream of JB comprehensively. Previous studies found that Meniere's disease might be related to the IJV outflow obstruction, but, regrettably, they did not pay attention to the connection between IJVS and high JB.^11^ And previous examinations were mainly based on temporal bone computed tomography, which could not detect IJVS adequately. Second, we evaluated bilateral JBs simultaneously by considering the dominant cerebral venous drainage, which was common and critical for cerebral venous diseases.^35^ Conversely, previous studies usually separated bilateral JBs and treated them equally to study their relationship with ear diseases.^3^, ^28^ The method might overlook the underlying pathogenesis associated with cerebral venous hemodynamics. Additionally, we studied high JB formation with each patient being a statistical unit, which was feasible to adjust for confounders in multivariate analysis. The high JB was reported to be more common in females, and patients usually had a varying span of onset‐to‐door time by the subacute or chronic clinical course.^28^ Adjusting for confounders would help to test the reliability of the results.

This study had several limitations. Firstly, the variants, such as diverticulum and dehiscence, of high JB were not further classified. However, focusing on the imaging features of high JB at the early phase and measuring its height quantitatively might contribute to discovering factors about the early formation of high JB for disease prevention. The relationship between venous stenosis and variants of high JB remains further studied. Secondly, the diagnosis in this study was based on CTV, which is radioactive and might limit its widespread applications. However, CTV is necessary for patients with suspicious high JB due to its close relationship with surrounding osseous structures. Applying radiation‐free examinations, such as MRV, for preliminary screening would help to avoid unnecessary CTV scans. Additionally, the causal relationship between IJVS and/or TSS and high JB is not settled due to the retrospective nature and the small sample size in this study, although the postnatal development theory and osseous structures surrounding JB support an external force to promote high JB formation. The findings in this study need to be interpreted with caution in the context of the study design because the level of evidence was low. Further prospective, multicenter studies with larger sample sizes and long‐term follow‐up are needed to confirm our findings.

## 5 CONCLUSION

This study, for the first time, proved the correlation between IJVS and high JB, which illustrated a possible mechanism for high JB formation in adult patients. IJVS and asymmetric transverse sinus were independently and positively associated with high JB, especially dominant‐side IJVS with dominant‐side high JB, indicating a potential hemodynamic relationship between IJVS and high JB formation. Conversely, TSS might impede high JB formation. Consequently, the venous stenosis up‐ and downstream of JB should be considered simultaneously when studying the pathogenesis and designing treatment strategies for diseases associated with high JB.

## AUTHOR CONTRIBUTIONS

Zhongao Wang wrote the first draft of the manuscript; Zhongao Wang, Yibing Guo, Meini Gao, Zixiang Wang, Duo Lan and Liqun Pan performed the material preparation, data collection and statistical analysis; Da Zhou, Ran Meng and Xunming Ji contributed to imaging assessments; Ran Meng, Chaitu Dandu and Yuchuan Ding wrote sections of the manuscript and contributed to manuscript revision; Xunming Ji and Ran Meng contributed conception and design of the study; Ran Meng takes full responsibility for the data, the analyses and interpretation, and the conduct of the research. All authors read and approved the submitted version.

## FUNDING INFORMATION

This work was supported by the Beijing Natural Science Foundation [grant number 7212047], and the National Natural Science Foundation of China [grant numbers 82171297, 82101390].

## CONFLICT OF INTEREST STATEMENT

The authors declare that there is no conflict of interest.

## Supporting information


**Fig. S1.** Forest plot of the potential risk factors for the features of high JB in the subset of patients with high JB exclusively. Panels A: potential risk factors for the presence of dominant‐side high JB. Panels B: potential risk factors for the presence of bilateral high JB. BMI, body mass index; CI, confidence interval; IJVS, internal jugular venous stenosis; JB, jugular bulb; OR, odds ratio; TSS, transverse sinus stenosis.

Table S1.

## Data Availability

The data that support the findings of this study are available from the corresponding author upon reasonable request.
