# Supplementary figures and images for: High jugular bulb in patients with non‐thrombotic internal jugular venous and transverse sinus stenosis: Clues to pathogenesis
Source: CNS Neurosci Ther. 2023 Aug 29;30(3):e14424. doi: 10.1111/cns.14424 (PMC10915994; doi:10.1111/cns.14424)

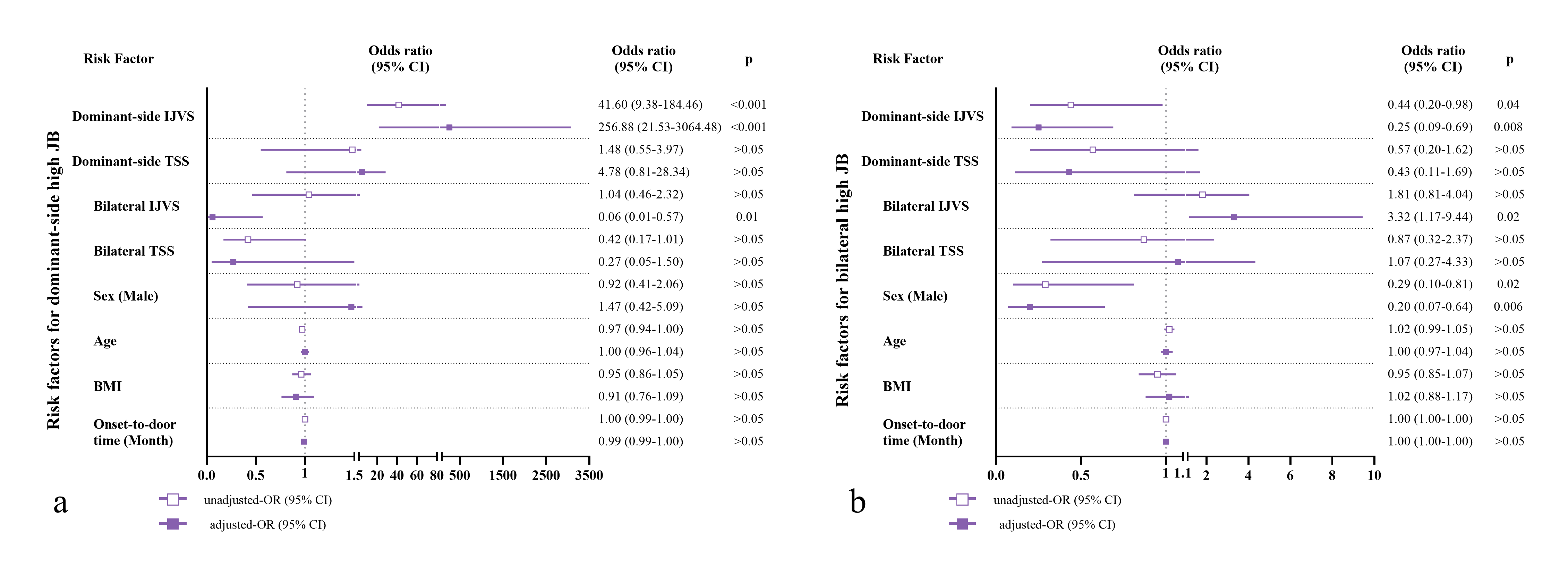

Supplement: Supplementary file 1 — Fig. S1. Forest plot of the potential risk factors for the features of high JB in the subset of patients with high JB exclusively. Panels A: potential risk factors for the presence of dominant‐side high JB. Panels B: potential risk factors for the presence of bilateral high JB. BMI, body mass index; CI, confidence interval; IJVS, internal jugular venous stenosis; JB, jugular bulb; OR, odds ratio; TSS, transverse sinus stenosis. [file CNS-30-e14424-s001.tif]
