# Supplementary material for: High jugular bulb in patients with non‐thrombotic internal jugular venous and transverse sinus stenosis: Clues to pathogenesis
Source: CNS Neurosci Ther. 2023 Aug 29;30(3):e14424. doi: 10.1111/cns.14424 (PMC10915994; doi:10.1111/cns.14424)
Supplement: Supplementary file 2 — Table S1. [file CNS-30-e14424-s002.docx]

**Table S1** Differences of clinical features between patients with high JB and non-high JB

|  | High JB  (n=142) | Non-high JB  (n=86) | p |  |
| --- | --- | --- | --- | --- |
| **Demographics** | | | |  |
| Sex（male）(No., %) | 48 (33.8) | 37 (43.0) | > 0.05 |  |
| Age (years) (Mean±SD) | 49.85±13.50 | 50.83±16.15 | > 0.05 |  |
| BMI (kg/m^2^) (Mean±SD) | 24.53±3.69 | 25.02±2.84 | > 0.05 |  |
| Onset-to-door time (months) (Median, IQR) | 18 (5-63) | 24 (6-51) | > 0.05 |  |
| **Clinical symptoms (No., %)** | | | |  |
| Headache | 70 (49.3) | 34 (39.5) | > 0.05 |  |
| Tinnitus | 61 (43.0) | 37 (43.0) | > 0.05 |  |
| Head noise | 60 (42.3) | 38 (44.2) | > 0.05 |  |
| Sleep disturbance | 60 (42.3) | 34 (39.5) | > 0.05 |  |
| Dizziness | 53 (37.3) | 32 (37.2) | > 0.05 |  |
| Visual impairment | 52 (36.6) | 28 (32.6) | > 0.05 |  |
| Hearing impairment | 24 (16.9) | 13 (15.1) | > 0.05 |  |
| Dry or puffy eyes | 17 (12.0) | 11 (12.8) | > 0.05 |  |
| Vertigo | 13 (9.2) | 5 (5.8) | > 0.05 |  |
| Double vision | 6 (4.2) | 6 (7.0) | > 0.05 |  |
| **Past medical history (No., %)** | | | | |
| Hypertension | 40 (28.2) | 34 (39.5) | > 0.05 |  |
| Diabetes | 12 (8.5) | 11 (12.8) | > 0.05 |  |
| Hyperlipemia | 46 (32.4) | 32 (37.2) | > 0.05 |  |
| Hyperhomocysteinemia | 7 (4.9) | 4 (4.7) | > 0.05 |  |
| Hyperuricemia | 8 (5.6) | 1 (1.2) | > 0.05 |  |

Abbreviations: JB, jugular bulb; BMI, body mass index; SD, standard deviation; IQR, inter quartile range.
